# Analysis of the genome of the New Zealand giant collembolan (*Holacanthella duospinosa*) sheds light on hexapod evolution

**DOI:** 10.1186/s12864-017-4197-1

**Published:** 2017-10-17

**Authors:** Chen Wu, Melissa D. Jordan, Richard D. Newcomb, Neil J. Gemmell, Sarah Bank, Karen Meusemann, Peter K. Dearden, Elizabeth J. Duncan, Sefanie Grosser, Kim Rutherford, Paul P. Gardner, Ross N. Crowhurst, Bernd Steinwender, Leah K. Tooman, Mark I. Stevens, Thomas R. Buckley

**Affiliations:** 1 Landcare Research, Private Bag, Auckland, 92170 New Zealand; 2 School of Biological Sciences, The University of Auckland, Auckland, New Zealand; 3 The New Zealand Institute for Plant & Food Research Ltd, Auckland, New Zealand; 4 Department of Anatomy, School of Biomedical Sciences, University of Otago, Dunedin, New Zealand; 5 Center for Molecular Biodiversity Research, Zoological Research Museum Alexander Koenig, Adenauerallee 160, 53113 Bonn, Germany; 6 Evolutionary Biology & Ecology, Institute for Biology, University of Freiburg, Freiburg, Germany; 7 Genetics Otago, Department of Biochemistry, University of Otago, Dunedin, New Zealand; 8 School of Biology, Faculty of Biological Sciences, University of Leeds, Leeds, LS2 9JT UK; 9 Department of Animal Behaviour, Bielefeld University, Bielefeld, Germany; 10 Division of Evolutionary Biology, Faculty of Biology, Ludwig-Maximilian University of Munich, Planegg-, Martinsried, Germany; 11 Biomolecular Interactions Centre, School of Biological Sciences, University of Canterbury, Christchurch, New Zealand; 12 South Australian Museum, North Terrace, GPO Box 234, Adelaide, SA 5001 Australia; 13 School of Pharmacy and Medical Sciences, University of South Australia, Adelaide, SA Australia

**Keywords:** Hexapoda, Neanuridae, Genome assembly, Phylogenomics, Methylation, Epigenetics, Developmental biology, RNA, Chemoreceptors, Sex determination, Horizontal gene transfer

## Abstract

**Background:**

The New Zealand collembolan genus *Holacanthella* contains the largest species of springtails (Collembola) in the world. Using Illumina technology we have sequenced and assembled a draft genome and transcriptome from *Holacanthella duospinosa* (Salmon). We have used this annotated assembly to investigate the genetic basis of a range of traits critical to the evolution of the Hexapoda, the phylogenetic position of *H. duospinosa* and potential horizontal gene transfer events.

**Results:**

Our genome assembly was ~375 Mbp in size with a scaffold N50 of ~230 Kbp and sequencing coverage of ~180×. DNA elements, LTRs and simple repeats and LINEs formed the largest components and SINEs were very rare. Phylogenomics (370,877 amino acids) placed *H. duospinosa* within the Neanuridae. We recovered orthologs of the conserved sex determination genes thought to play a role in sex determination. Analysis of CpG content suggested the absence of DNA methylation, and consistent with this we were unable to detect orthologs of the DNA methyltransferase enzymes. The small subunit rRNA gene contained a possible retrotransposon. The *Hox* gene complex was broken over two scaffolds. For chemosensory ability, at least 15 and 18 ionotropic glutamate and gustatory receptors were identified, respectively. However, we were unable to identify any odorant receptors or their obligate co-receptor Orco. Twenty-three chitinase-like genes were identified from the assembly. Members of this multigene family may play roles in the digestion of fungal cell walls, a common food source for these saproxylic organisms. We also detected 59 and 96 genes that blasted to bacteria and fungi, respectively, but were located on scaffolds that otherwise contained arthropod genes.

**Conclusions:**

The genome of *H. duospinosa* contains some unusual features including a *Hox* complex broken over two scaffolds, in a different manner to other arthropod species, a lack of odorant receptor genes and an apparent lack of environmentally responsive DNA methylation, unlike many other arthropods. Our detection of candidate horizontal gene transfer candidates confirms that this phenomenon is occurring across Collembola. These findings allow us to narrow down the regions of the arthropod phylogeny where key innovations have occurred that have facilitated the evolutionary success of Hexapoda.

**Electronic supplementary material:**

The online version of this article (10.1186/s12864-017-4197-1) contains supplementary material, which is available to authorized users.

## Background

Collembola (springtails) are an ancient group within Hexapoda, with extinct species known from the Palaeozoic [1] and molecular dating analyses suggesting a divergence from their sister taxon in the Ordovician to Devonian [2]. The existence of Collembola at such an early point in the evolution of terrestrial life indicates that they made up an important component of the earliest terrestrial ecosystems, with the group today found in almost all ecosystems on earth including those on Antarctica [3]. Given their ecological ubiquity and phylogenetic position, understanding the genetic basis of Collembola’s key traits is crucial to understanding their success and that of more derived hexapod groups such as ectognathous insects. The placement of Collembola within the arthropods is a particular problem that morphological analyses [4, 5] and complete mitochondrial genome sequences (see [6]) have failed to conclusively resolve, with efforts now shifting to analysis of whole genomes and transcriptomes (e.g., [2, 7–10]). Resolving the placement of Collembola would allow a better understanding of the origins and evolution of Insecta, the colonisation of land by arthropods and the evolution of key traits within Collembola and more generally across Hexapoda.

One of the most specialised groups of Collembola are part of the hyperdiverse saproxylic communities that drive log decay and nutrient cycling and thereby assist in nutrient uptake by plants in forests by returning nutrients from dead wood to the ecosystem [11–13]. In New Zealand Uchidanurinae Salmon, 1964 (Collembola: Neanuridae) are a particularly important part of the saproxylic fauna [14, 15]. The subfamily currently consists of five endemic New Zealand species *Holacanthella spinosa* Lubbock, *H. paucispinosa* Salmon, *H. brevispinosa* Salmon, *H. laterospinosa* Salmon and *H. duospinosa* Salmon and are unusually large in size (up to 17 mm) possessing brightly coloured digitations (epidermal spine-like projections) on their dorsal and lateral surfaces [15].

Recently two genome assemblies from Collembola have been published; *Orchesella cincta*, from the family Entomobryidae [10] and *Folsomia candida* from the family Isotomidae [16]. Analysis of both of these genomes demonstrated a large number of horizontal transfer events from bacteria and fungi, as well as differential gene family expansions associated with adaptation to environmental stresses. Whole genome sequencing and transcriptome sequencing, either in conjunction or separately, have proven informative in revealing the genomic basis of key traits in arthropods [17–25]. Despite these significant insights into collembolan biology there are a number of unanswered questions. First, the species *O. cincta* and *F. candida* both inhabit soil environments. Other collembolan taxa such as *Holacanthella* inhabit leaf litter and dead wood, which are very different environments and likely to place very different selective pressures on genome evolution. Analysis of further collembolan genomes are required to elucidate the effects of these different lifestyles. Second, there are a number of critical evolutionary transitions in hexapod evolution for which the role of Collembola is currently unknown. Despite much research on the evolution of sex determination in insects, very little is known about how this occurs in Collembola. Furthermore, the presence of many key arthropod sex determination genes in Collembola has yet to be established [26]. There has been recent attention to the evolution of DNA methylation and associated enzymes within Insecta [27], however the earlier diverging hexapods have yet to be fully examined for DNA methylation and the presence of the key DNA methylation enzymes. Likewise, understanding the suite of chemoreception and chitinase proteins in Collembola is critical for understanding the evolution of associated traits in higher insects and their potential role in driving the diversification of terrestrial arthropods.

Here we have generated a draft, annotated genome assembly for the New Zealand giant collembolan, *Holacanthella duospinosa*. We use a combination of genome sequence, transcriptomic data and annotations to infer the genetic basis of key traits within Collembola. Our genomic resources shed light on the evolution of several key innovations within the Hexapoda, including the genetic basis of sex determination, key development pathways, DNA methylation, chemoreception, and chitinase activity, thereby providing a resource for the further study of hexapod evolution.

## Results and discussion

### De novo genome and transcriptome assemblies

Our genome assembly has a total size of 375 Mbp, constructed from ~72 Gb of genomic raw sequence reads (Table 1) with 2.18% of sites as missing data. This compares to an estimate of 320 Mbp from flow cytometry. The N50 is 230,133 bp, with a maximum scaffold length of 2.8 Mbp (Table 2). The DNA and RNA-seq mapping rates were 99.8% and 82%, respectively. The percentage of TBLASTN matches to microbes was approximately 0.2%, indicating very low levels of DNA contamination or horizontal gene transfer (see below). These results, together with the high level of complete genes (95.3%) recovered from comparison with the BUSCO v2.0.1 database ([28], arthropoda_odb9), suggests a high quality genome assembly suitable for annotation and analysis. The transcriptome assembly (Table 3) included 152,441 contigs with a N50 of 2129 bp. Contig lengths ranged from 101 bp to 24,141 bp.

**Table 1:** Sequencing output used to assemble the *Holacanthella duospinosa* genome

Insert size	Sequencing output (Gb)	Number of reads	Genome coverage (X)
188 bp	26.9	266,061,330	84.1
200 bp	6.9	68,600,986	21.6
470 bp	34.8	344,690,702	108.8
3 kb	1.9	185,408,672	5.9
5 kb	1.5	143,938,120	4.7
Total	72	1,008,699,810	225.1

**Table 2:** Summary of the *Holacanthella duospinosa* genome assembly

	Size (bp)	Number
N90	147	103,690
N80	3137	5801
N70	17,443	1588
N60	83,545	567
N50	226,503	317
Total (>100 bp)	370,315,149	410,937
Total (>2 kb)	299,867,363	8059
Longest (bp)	2,807,427	
GC (%)	33.40	
N (%)	2.18

**Table 3:** Summary of the *Holacanthella duospinosa* transcriptome assembly

	Transcriptome assembly
Total (bp)	108,127,906
Number	152,441
N50 (bp)	2129
Shortest (bp)	101
Longest (bp)	24,141
Mean (bp)	709
Median (bp)	234
Number of contigs >500 bp	44,149
Number of contigs >1000 bp	27,986
Number of contigs >10 k bp	183
GC%	36.25

### Annotation of structural genes, repeats and horizontal gene transfer

Analysis of repeats yielded 3182 repeat models, from which we discarded 40 as they had significant similarity (e-value 10^−5^) to some protein-coding genes from the NCBI non-redundant database. Of the remaining repeat models 571 were able to be classified into known repeat groups. This allowed us to mask 42.96% of the genome assembly with these repeat models (Table 4). The class I transposable elements (TEs), including long terminal repeat retrotransposon (LTR), non-LTR long interspersed retrotransposon (LINE) and short interspersed retrotransposon (SINE), formed only 4.37% of the genome, in contrast with larger hexapod genomes (e.g., [23]). Among these repeats, LTRs comprised 2.78% of the genome assembly with the most abundant family being Gypsy, which corresponded to 27,612 copies of the element, making up 2.0% of the genome. The Gypsy repeat, rnd.-1_famiy-178, had the greatest number of copies, indicating it has been highly active in the evolution of Collembola. The most abundant LINE family was CRE-II, containing 4862 copies, comprising over 1.2 Mbp of genomic DNA. SINEs were rare, with only 12 incomplete SINE/SINE-like fragments detected. The class II DNA elements comprised 8.42% of the genome representing the most abundant repeat class in the assembly. The family TcMar-Tc1 had the greatest copy number (21,888) making up 1.87% of the genome, among which, rnd.-1_family-48 was the most frequent TcMar-Tc1 family found within the assembly.

**Table 4:** Comparison of repeat components between *Holacanthella duspinosa* and *Drosophila melanogaster* genomes

	*H. duospinosa*		*D. melanogaster*	
Types	Length (bp)	P%	Length (bp)	P%
DNA	31,620,408	8.42	4,849,763	2.87
LINE	5,971,075	1.59	12,119,904	7.18
LTR	10,439,992	2.78	21,849,378	12.95
SINE	110,785	0.00	52,841	0.03
Simple repeat	6,196,398	1.65	2733	0.00
Other	640,294	0.17	698,554	0.41
Unknown	106,352,725	28.32	11,211,970	6.64
Total	161,336,129	42.96	50,785,143	30.00

**Fig. 1:**
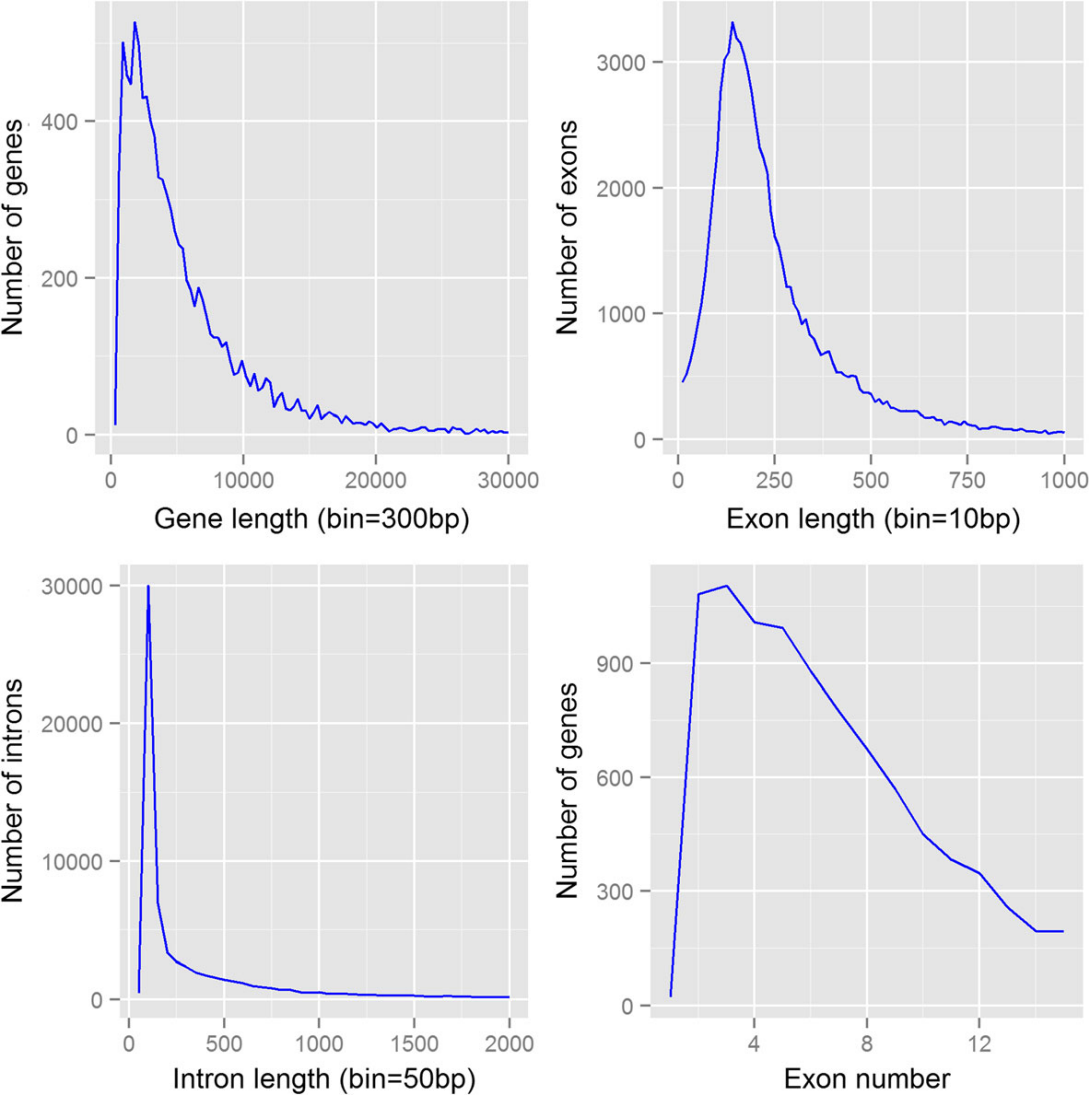
Distribution of gene parameters for the genome assembly of *Holacanthella duspinosa*

The genome annotation generated 12,000 gene models, of which 9911 were supported and revised by homologous sequences. Of this highly confident set of 9911 gene models, the average gene length was 5733 bp with an average intron number and length of seven and 547 bp, respectively; introns were shorter than in many published insect genomes (e.g., [23]). The distribution of these gene parameters across the genome is given in Fig. 1. Of the 1066 conserved BUSCO genes, 825 (77.4%) of this gene/protein set were predicted to be full length and 69 (6.5%) partial. Among the complete genes, most of them represent single copies (90.4%). We then annotated the protein models from comparisons with the NCBI Genbank non-redundant (nr) database using BLASTP with a cut-off threshold of e-value 10^−6^. The proportion of protein models showing homology to nr records was 82.8% and the two species that the proteins hit most frequently were *Zootermopsis nevadensis* (6.0%) and *Daphnia pulex* (3.6%) (Additional file 1). Both of these analyses demonstrate that this set of predicted gene models is largely representative of the *H. duospinosa* protein coding sequences, and is therefore suitable for subsequent evolutionary and functional studies.

**Fig. 2:**
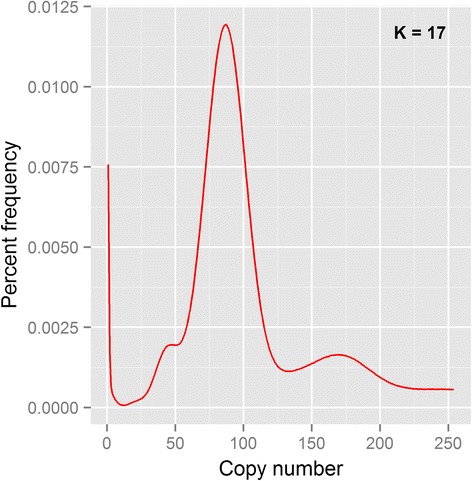
Kmer spectrum for the genome assembly of *Holacanthella duspinosa*

The total level of heterozygosity within the *H. duospinosa* genome, which is the portion of heterozygous single-nucleotide polymorphisms between the two haploid components in the diploid genome, was estimated to be 1.56 × 10^−3^. Among all called variants, including indels, 20,622 (2.97%) fell within the coding regions of 6150 annotated gene models in 13,162 exons (Additional file 2). The histogram of k-mer copy number was largely uni-modal, reflecting the low level of heterozygosity (Fig. 2).

We identified a total of 59 bacterial and 96 fungal genes as candidates for horizontal gene transfer (HGT) into the *H. duospinosa* genome (Additional file 3). Compared with the *Folsomia candida* [16] and *Orchesella cincta* [10] genomes, we have found fewer candidate HGTs, which is likely due to the *H. duospinosa* genome being assembled from short Illumina reads and therefore being highly fragmented (Table 2). Nonetheless, the most common blast hits of the HGT candidates are from the two fungal species (*Conidiobolus coronatus* and *Basidiobolus meristosporus*). The HGT candidates are involved in a wide variety of metabolic functions, like those identified from *Folsomia candida* and *Orchesella cincta* [10, 16]. These include amino acid production, DNA and glycerol metabolic process, ATP synthesis, oxidation-reduction process and cation transport. Our fragmented assembly, along with non-curated genes models, have likely led to an underestimate of the amount of HGT into the *H. duospinosa* genome. However, our results do confirm that this process is a general one within Collembola.

### DNA methylation

**Fig. 3:**
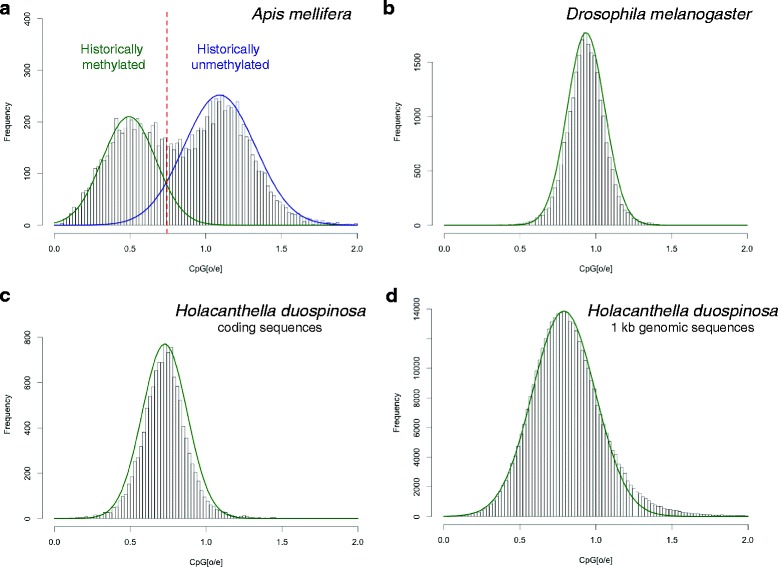
Signatures of normalised CpG content (CpG[o/e]) reveal the presence and absence of historical DNA methylation in hexapods. Graphs are frequency histograms of CpG[o/e] with the y-axis depicting the number of genes with the specific CpG[o/e] values given on the x-axis. **a** Analysis of gene bodies in the honeybee (*Apis mellifera*), which has an intact DNA methylation system, reveals a bimodal distribution. **b** In contrast, the same analysis in *Drosophila melanogaster*, which does not have an intact DNA methylation system, reveals a unimodal distribution. **c** Analysis of *Holacanthella duospinosa* transcripts reveals a similar unimodal distribution consistent with the absence of an intact DNA methylation system in this species. The mean of this distribution is similar to the mean obtained for 1 kb fragments of the genome (**d**) and is consistent with a slightly lower than expected CpG content in the DNA sequence of *H. duospinosa*

In arthropods DNA methylation (the addition of a methyl group to a cytosine residue in a CpG context) occurs predominantly within the exons and introns of genes [29–31]. Methylation of cytosine residues leaves them susceptible to deamination [32] and, over evolutionary time, genes that are highly methylated (in germ-line cells) will have lower than expected CpG content. This affect can be quantified by calculating the normalised CpG content of genes, or CpG[o/e]. In animals where DNA methylation has a demonstrated role in controlling gene expression, such as *Apis mellifera* (Fig. 3a), the distribution of CpG[o/e] values can be described as, consisting of genes with lower than expected CpG content that are historically methylated and those with higher than expected CpG content that are historically unmethylated. Predictions of historical DNA methylation using this method correlate with measured levels of DNA methylation [33, 34]. In contrast, the same analysis in *Drosophila melanogaster,* which does not have an intact DNA methylation system and has very low levels of DNA methylation [35, 36], yields a unimodal distribution (Fig. 3b). Analysis of the CpG content of genes predicted from the *H. duospinosa* genome displayed a unimodal distribution indicating the absence of historical DNA methylation in this species. The mean CpG[o/e] is 0.7, which is lower than the expected value of one but consistent with the relatively low CpG content of the *H. duospinosa* genome as a whole (mean CpG[o/e] is 0.79).

A full complement of the DNA methyltransferase enzymes, *Dnmt1*, *Dnmt2* (*TRDMT1*) and *Dnmt3*, is thought to be required for a fully functional DNA methylation system [37]. *Dnmt3* enzymes are the ‘de novo’ methyltransferases and are important in mediating environmentally responsive DNA methylation [37]. *Dnmt2* (*TRDMT1*) methyltransferases are predominantly involved in tRNA methylation and *Dnmt1* DNA methyltransferases act as maintenance methyltransferases maintaining methylation marks across cell division [37]. The *H. duospinosa* genome encodes three orthologs of the tRNA methyltransferase *Dnmt2* and an ortholog of the putative DNA demethylation enzyme *Tet1* [38, 39], an enzyme that also functions in the modification of mRNA promoting translation in *D. melanogaster* [40]. However we were unable to identify an ortholog of the de novo methyltransferase, *Dnmt3* or the maintenance methyltransferase *Dnmt1*, within the *H. duospinosa* genome. The lack of *Dnmt1* and *Dnmt3* in *H. duospinosa* is consistent with the absence of any environmentally responsive DNA methylation in this organism predicted from the analysis of CpG[o/e].

### Non coding RNA genes

Non-coding RNAs (ncRNAs) form a central role in the catalysis and regulation of key cellular functions such as translation, splicing, transport and the modulation of gene expression. The major RNA families include essential and highly conserved RNAs such as the tRNAs, rRNAs and the RNA components of RNase P and the signal recognition particle [41]. Other ncRNAs, such as the small nucleolar RNAs (snoRNAs), microRNAs (miRNAs) and the long non-coding RNAs (lncRNAs), have a high evolutionary turnover [42, 43]. The ncRNAs pose serious research challenges for genome annotation as they lack many of the strong statistical signals that are associated with protein-coding genes, such as open reading frames and codon-usage biases, and are frequently pseudogenised and duplicated via transposition [44]. Therefore homology-based approaches, as opposed to *de-novo* prediction, are generally used to find them, although high throughput transcriptomic approaches are increasingly employed [45].

The essential and well conserved metazoan ncRNAs: tRNAs, rRNAs (*5S*, *5.8S*, *SSU* and *LSU*), *RNase P*, *RNase MRP*, *SRP* and the major spliceosomal snRNAs (*U1*, *U2*, *U4*, *U5*, *U6*), as well as the minor spliceosomal snRNAs (*U11*, *U12* and *U6atac*), were all found in the *H. duospinosa* genome assembly. Only the *U4atac* component of the minor spliceosomal snRNAs is missing. The copy number of the *serine tRNA* is relatively high (548, the average is 18 for the other 19 canonical amino-acid accepting tRNAs). Many of these are likely to be SINEs derived by transposition including those that were not predicted from the de novo repeat modelling approach. All the 20 tRNA isotypes were identified in the assembly. Again, many of these had rather large copy numbers (Table 5), ranging from 5 (Trp) to 548 (Ser).

**Table 5:** The genomic copy numbers of the transfer RNA isotypes predicted by tRNAscan and Rfam. The Rfam predictions that did not overlap with tRNAscan predictions are in parentheses

Isotype	Copy number
Ala	21
Arg	26
Asn	9
Asp	9
Cys	11
Gln	11
Glu	13
Cly	17
His	6
Ilse	17
Leu	28
Lys	19
Met	15
Phe	18
Pro	65
Ser	548
Sup	1
Thr	21
Trp	5
Tyr	12
Val	20
Pseudo	69
SeC	6
Undetermined	9 (+374)

We identified 17 loci with sequence similarity to nine known snoRNA families. These included one scaRNA (*SCARNA8*), three H/ACA box and 13 C/D box snoRNA associated loci. The snoRNAs are predominantly involved with rRNA maturation. We identified 20 loci with sequence similarity to 14 microRNA families. A number of cis-regulatory elements were also identified. These include 118 *histone 3′ UTR* stem-loops, three potassium channel RNA editing signal sequences, four selenocysteine insertion sequences (SECIS) and three internal ribosome entry sites (IRES).

The predicted *SSU rRNA* on “scaffold300_size451797/208930–205,788” matches NCBI-nr sequences from the collembolan species, *Morulina verrucosa* (Neanuridae: Morulininae) and *Crossodonthina koreana* (Neanuridae: Neanurinae). However, there is a large, 1454 bp insertion in the *SSU rRNA* at position 496 to 1949. This region contains a homolog of a reverse transcriptase, suggesting that this rRNA insert is a retrotransposon. We detected eight paralogues of this insertion sequence in the *H. duospinosa* genome and transcriptome sequences.

### Developmental genes

**Fig. 4:**
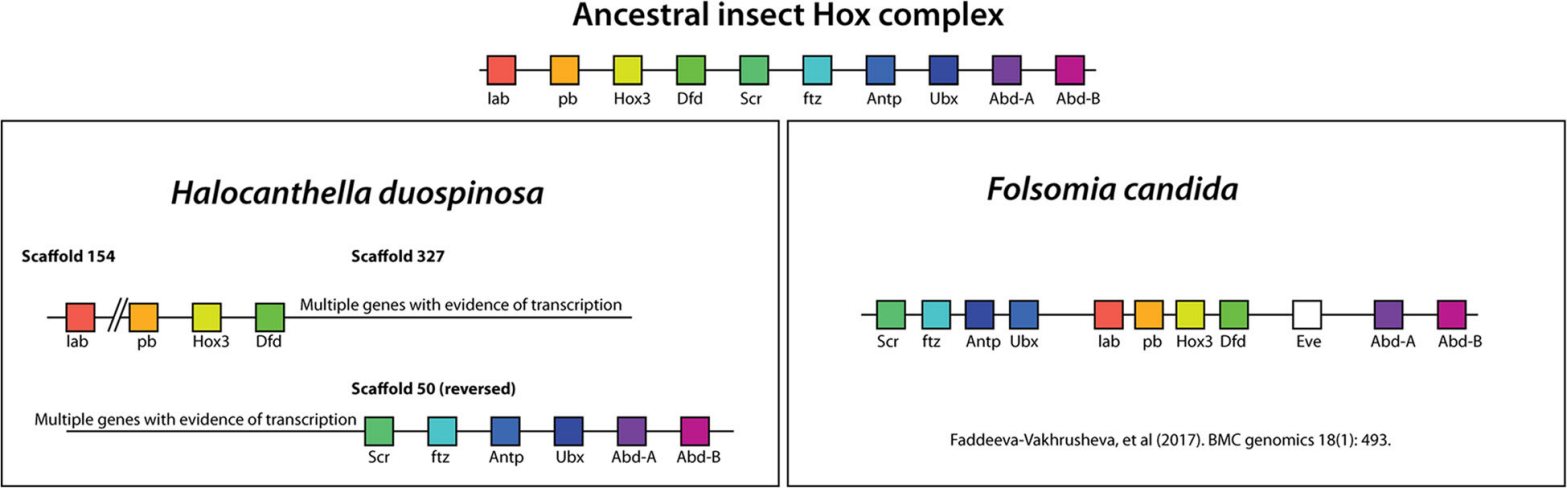
Arrangement of the Hox gene cluster in *Holacanthella duospinosa* relative to the collembolan *Folsomia candida* and a hypothetical ancestral insect

Axis formation genes evolve relatively rapidly in insect lineages [46], and patterns of loss and conservation are well known. *Holacanthella duospinosa* has no Bicoid, which despite being a key gene in *Drosophila*, is restricted to dipteran lineages. *Holacanthella duospinosa* is also missing, like most non-dipteran insects, classical oskar and swallow genes. Genes involved in terminal patterning are well conserved, with a gene related to trunk, as well as a noggin-like gene [47], both present in the *H. duospinosa* genome. The genes that control segmentation in insects are generally well conserved. The Hox gene complex is an evolutionarily conserved complex of homeobox containing genes derived from the common ancestor of metazoans. The genes in the complex control segmental identity and their duplication and diversification have been instrumental in the evolution of the metazoan body plan [48, 49]. The relationships of genes in the complex, their order along the chromosome and transcriptional direction are all highly conserved. In *H. duospinosa*, the Hox gene complex is broken over two genome regions. At the 3′ end of the complex, The gene labial is found at the extreme end of scaffold 154. This is likely linked to the next region of the complex, found on scaffold 327, which contains three genes, proboscipedia, hox3 and Deformed. The rest of this scaffold contains multiple genes with strong similarity to evolutionary conserved sequences and evidence of transcription. The 5′ end of the complex (Sex-combs reduced, fushi-taratzu, Antennapedia, Ultrabithorax, abdominal-A and Abdominal-B) are found on scaffold 50. At the 3′ end of this group of genes are multiple genes with strong similarity to evolutionary conserved sequences and evidence for transcription. This genomic structure implies the Hox gene complex is split in *H. duospinosa* (Fig. 4), which is unusual in insects, but is found especially in Diptera. The alternative possibility is that this is an assembly error, though the placement of conserved, transcribed genes at the ends of the contigs containing both parts of the assembly suggest otherwise. The apparent split in the Hox gene cluster of *H. duospinosa* is at a different position to those found in *Drosophila* species [50–52] or in the silkworm, *Bombyx mori* [53]. The split in the Hox complex described here is also partially consistent with the rearrangement seen in the genome of the collembolan *Folsomia candida* [16], where a significant complex inversion separates deformed from sex-combs reduced, placing sex combs nearer *Abd-A* and splitting *AbdA* from *Ubx*. In *Folsomia*, deformed and sex combs reduced are on different scaffolds, but *Ubx* and *AbdA* are conventionally arranged next to each other (Fig. 4).

Notch signalling is a highly conserved animal-specific cell-signalling pathway with little change observed over evolutionary time. In *H. duospinosa*, most of the pathway is conserved. Surprisingly, however, orthologs of Deltex and Serrate were not found in the assembly. These two genes are core components of the Notch pathway conserved in all other insects we have looked at, suggesting either that the genome assembly is incomplete or that there has been lineage-specific loss of these genes. Given that Notch signalling is a pleiotropic pathway with many roles in development and in adult tissues it is not clear what the consequence of the loss of these genes might be.

The Enhancer of split complex is an unusual gene complex found in insects and Crustacea that consists of bHLH-orange domain genes and bearded class genes. This gene complex is Notch signalling responsive [54, 55], and the genes in the complex encode Notch effector proteins [56]. Ancestrally, the complex is made up of four genes, three bHLH-orange genes and a bearded class gene, with this structure conserved (with variation) in insects and Crustacea [57, 58]. In *H. duospinosa*, the enhancer of split complex is reduced to two bHLH-orange genes (*her* and *bHLH1*) on scaffold 36. No bearded class gene is present in the complex; though others may be present in the genome (bearded class genes have little sequence similarity). Reductions in the Enhancer of split complex are common especially in hemipteran insects [57, 58], but it is not clear what effect this reduction might have on Notch-regulated processes.

The runt complex is an insect-specific gene complex [59] comprised of four runt domain encoding genes. The runt complex in *H. duospinosa* lies on scaffold 154 (upstream from the start of the Hox gene complex) and is identical to those found in other insects [59].

### Sex determination genes

**Fig. 5:**
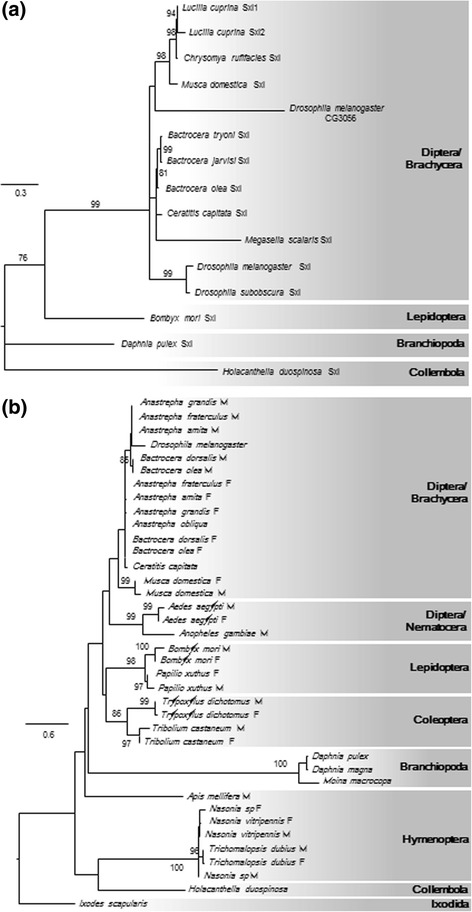
Phylogenetic trees of doublesex (**a**) protein sequences and Sex-lethal (**b**) protein sequences. F and M denote female and male splice variants, respectively. Numbers above branches are bootstrap proportions where only values greater than 50% are given. The scale bars show the expected number of amino acid substitutions per site

In hexapods, a large variety of molecular mechanisms have been described that determine the sexual fate of an individual (for reviews, see [26]). While a remarkable diversity of upstream components of the sex determination cascade have evolved within different hexapod groups, a few key regulatory genes are highly conserved among all taxa investigated to date. One of the key players is doublesex (*dsx*), a transcription factor belonging to the DM-domain family of genes, which are involved in sex-determination and sexual differentiation in all metazoans [60, 61]. In insects, *dsx* contains two conserved domains, a DNA binding domain (DM-domain; *dsx* and *mab-3*) and a dimerisation domain (Dimer) [62]. *Dsx* has been described as the master switch gene at the bottom of the sex-determination cascade in all insects. It undergoes sex-specific splicing by transformer (*tra*), which represents another key player in most, but not all insect sex-determining pathways [63]. Little is currently known about sex determination in Collembola and no molecular mechanisms have been described for this group. Expressed Sequence Tag data [64] provided evidence for *dsx* in Collembola, identifying both the DM and Dimer domains and a potential alternative splicing of Dimer. However, both domains were only present as singletons on different contigs. Here we identified a putative *H. duospinosa dsx* transcript of 485 amino acids that contains both, a DM and Dimer domain (Fig. 5a, Additional file 4). No homologues were found for *tra*, however this gene can be highly divergent among insect lineages [65], which limits our ability to detect *tra* based on sequence similarity. We did, however, identify a putative transformer 2 transcript, which, in *Drosophila*, forms a complex with *tra* to control the sex-specific splicing of *dsx* pre-mRNA.

Sex-lethal (*Sxl*) is one of the master regulatory genes in drosophilid sex-determination and is thought to have co-opted its specific function as a result of a gene duplication event in the fly clade [66]. In *H. duospinosa* we likely detected the *Sxl* paralogue, CG3056 or sister-of-sex-lethal (Fig. 5b, Additional file 5). It is unclear whether this gene plays a role in sex-determination in insects. Other putative sex-determining genes detected in *H. duospinosa* are listed in Additional file 6.

Overall, we found many transcripts with sequence similarities to genes that, in insects, are involved in responses to X:Autosome signals, in dosage compensation, and processing of doublesex and Sex-lethal (Additional file 6). It is unclear whether in *H. duospinoasa* these genes are involved in sex-determination, or if these specific functions were co-opted only later in hexapod evolution. Further experimental work would be needed to determine the exact pathway of sex determination in this species; but our data, together with data from other hexapods, are steadily building our understanding of the core features, and differences of this process, across this startlingly specious taxon.

### Chemosensory genes

Collembola are able to respond to odours and tastants, being repelled by bitter, alkaline, acid and salt tastes in food [67, 68]. Thought to have originated early in protostome evolution, ionotropic glutamate receptors (IRs) are involved in chemoreception in insects primarily detecting low volatility acids [69]. Using IRs from *Drosophila melanogaster* and *Dendroctonus ponderosae* (mountain pine beetle) as query sequences, at least 15 IRs were identified from the *H. duospinosa* genome assembly. They included orthologues of the IR co-receptors *IR25a* and *IR8a*, suggesting that this collembolan has a functional IR system. Candidate ligands might be feeding cues and pheromones involved in the location of potential mates or conspecifics.

Gustatory receptors (GRs) are involved primarily in taste reception in protostome invertebrates [70], however their cnidarian relatives have a role in pattern formation [71]. In the *H. duospinosa* genome assembly 18 GRs were identified using louse and termite GRs in BLAST searches with a cut-off of 1e^−05^. We also applied the rule that hits needed to contain a C terminal motif of T/SXXXXXXQF, where X = an aliphatic amino acid. No GRs involved in carbon dioxide sensing (*Gr21a* and *Gr63a*) were found, consistent with previous findings that carbon dioxide sensing GRs evolved later within insect evolution [72]. It is not clear what tastants the collembola GRs are capable of detecting, however likely candidates include compounds that are indicators of nutritional value and toxins (bitter compounds).

Likely derived from the GRs, the odorant receptors (ORs) are involved in odor reception and have undergone differential gene family expansion and are now a very large gene family in higher insects [73]. Recent genomic studies have suggested that ORs, or at least their obligate co-receptor, Orco, may have evolved early in hexapod evolution. The genomes of crustaceans do not contain Orco or any ORs [74] and ORs were not detected within the genome of a bristletail [75]. Orco has been, however, identified in the genome of a firebrat [75]. Using Orco sequences from locust and firebrat (*Thermobia domestica*) we could not find any evidence for an Orco orthologue within the Collembola genome or transcriptome. Similarly no other ORs were identified in searches using insect OR sequences with a cut-off of 1e^−05^. The lack of any odorant receptors in Collembola is consistent with the hypothesis that the expansion of these genes within insects has been associated with the evolution of insect flight [45].

### Chitinase genes

Collembola are members of the Ecdysozoa, a group of protostome metazoans that moult as they grow. The moulting process requires the ability to reshape the chitin that makes up their exoskeleton and chitinases are an important family of enzymes involved in this process. Chitinases may also play an important role in the degradation of fungal hyphae, a major food source for collembolans [76, 77]. Previous research has shown that Collembola display chitinase activity and are therefore able to digest fungal cell walls [77] and the *Orchesella cincta* genome shows a wide array of chitinase genes [10]. Twenty three chitinase-like genes were identified from the genome of *H. duospinosa*. Apart from three genes, *Cht2*, *Cht8* and *Cht10*, which were tandemly arranged on the same scaffold, the chitinase-like genes were identified within discrete scaffolds. Analysis of the transcriptome provided full transcripts for seven of these genes and partial transcripts for five. Evidence for at least one pseudogene was found. Twelve genes did not have any representative sequences in the transcriptome, perhaps indicating these genes could be expressed at different developmental stages not investigated here. *Holacanthella duospinosa* has a similar number of chitinase-like genes when compared with other crustaceans and insects, including *Daphnia*, *Drosophila*, beetles and mosquitos [21, 78].

Insect chitinase and chitinase-like proteins generally contain a combination of single or multiple chitin-binding domains and hydrolase domains, specifically from the glycoside hydrolase 18 (*GH18*) family. Here we have defined chitinases based on the presence of at least one chitin hydrolase domain. All the *H. duospinosa* hydrolyase domains fall into the *GH18* family, except *Cht23* which falls into the *GH19* family, predominantly restricted to plants. The pattern of *H. duospinosa* chitinase protein domain structures includes simple proteins with a single chitin hydrolase domain to the more complex *Cht3* that contains three hydrolase domains interspersed with four chitin binding domains. Orthologues of this gene are found in higher insects (eg. *Cht10* of *Tribolium*; [78]), and generally contain 4–5 domains of either type. The crustacean, *Daphnia pulex*, has an orthologue (*Cht3*) that contains the same number of domains as *H. duospinosa*, perhaps indicating an expansion of these domains has occurred in insects.

**Fig. 6:**
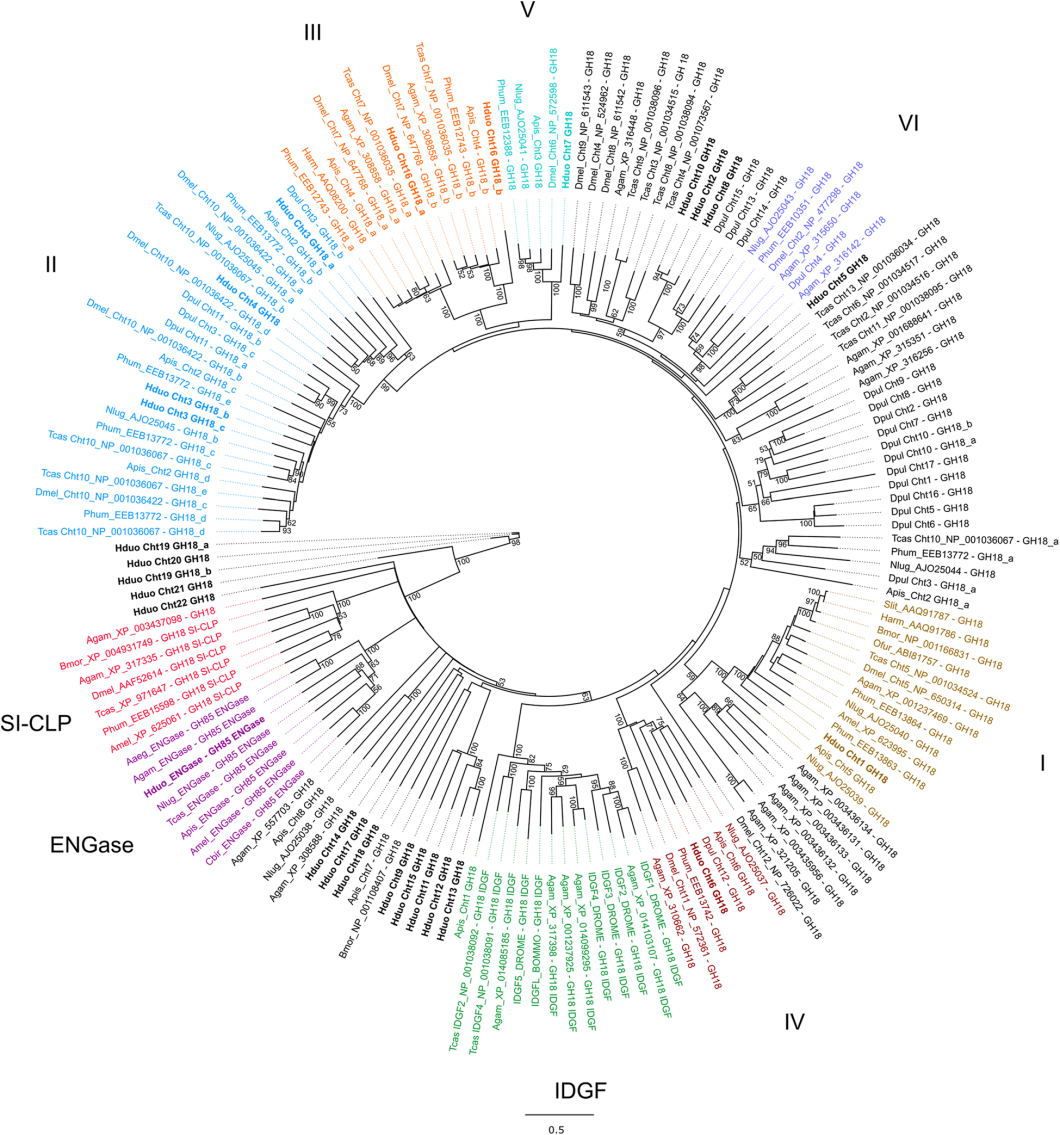
Maximum likelihood phylogenetic tree of the glycosyl hydrolase family 18 (*GH18*) domains from chitinase-like proteins of insects. Includes sequences from *Aedes aegypti* (Aaeg), *Anopheles gambaie* (Agam), *Apis mellifera* (Amel), *Acyrthosiphon pisum* (Apis), *Bombyx mori* (Bmor), *Cerapachys biroi* (Cbir), *Drosophila melanogaster* (Dmel), *Daphnia pulex* (Dpul), *Helicoverpa armigera* (Harm), *Holacanthella duospinosa* (Hduo), *Nilaparvata lugens* (Nlug), *Ostrinia furnacalis* (Ofur), *Pediculus humanus corporis* (Phum), *Spodoptera litura* (Slit), and *Tribolium castaneum* (Tcas). Values at the nodes are bootstrap support percentages over 50%. Chitinase-like proteins identified from *H. duospinosa* are indicated in bold. Classification of chitinase groups follows [75]

A phylogeny comprised of extracted *GH18* hydrolase domains was used to compare the chitinase-like proteins of *H. duospinosa* to those of crustaceans and insects (Fig. 6). The phylogeny displays conserved orthologous groups that include *GH18* domain sequences from *H. duospinosa* and a number of collembolan specific expansions. *Holacanthella duospinosa* has members of a number the conserved chitinase groups, including Group I, II, III, IV and V chitinases and ENGases of [79]. However, no obvious members of the SI-CLPs or IDGFs were identified from *H. duospinosa*. Four expansions including *H. duospinosa* sequences were identified (*Cht2*, *8*, *10*; *Cht9*, *11*, *12*, *13*, *15*; *Cht14*, *17*, *18* and *Cht19*, *20*, *21*, *22*) that have yet to be assigned to chitinase groups described in the literature. Of the *H. duospinosa* chitinase hydrolase domains all except *Cht3a*, *14*, *16b*, *19a*, *19b*, *20* and *21* contain a glutamic acid within the conserved motif II, synonymous with chitinolytic activity.

Chitinase genes have been shown to be differentially expressed throughout development of the insect. In this study, RNAseq data was collected from tissue taken from an adult, hence expression will be exclusive to adult physiology. The *H. duospinosa* chitinases with evidence for expression from RNAseq include *Cht1*–*8*, *10*, *22* and *23*. Group I and II chitinases have been shown to be involved in degradation of the endocutile during moulting [78]. Certainly *H. duospinosa* has orthologues within these two groups (*Cht1* and *Cht3*, respectively). Group III are anchored in the plasma membrane by a TM domain and are involved in processes post moulting [78]. *Holacanthella duospinosa* has an orthologue in this group also, *Cht16*, however it does not seem to be expressed in the adult. Collembola are members of the Ecdysozoa and therefore moult as they grow so having these conserved chitinases is anticipated. A recent study has implicated a role for *Drosophila Cht11* in regulation of cholesterol within mitochondria, impacting pathogen infection [80]. *Holacanthella duospinosa Cht6* falls into the same phylogenetic clade as *Cht11* from *Drosophila*, which may indicate a similar role for this chitinase from Collembola. Since fungi are thought to be a major part of collembolan diet it is conceivable that some of these chitinases also may be involved in the digestion of fungal cell wall material.

### Phylogenetic analysis based on transcriptome data

We assembled a large set of orthologous genes from which to reconstruct phylogenetic relationships among early diverging hexapod lineages. Of 1478 single copy orthologous genes [2], we found hits for more than one of the nine species for 1470 OGs (Additional file 7). The subsequent outlier check revealed no outlier sequences for any of the nine query species. The identification of protein domains revealed 4026 unannotated regions (so-called voids) and 2841 Pfam-A data blocks. After deleting the ambiguously aligned sections and concatenation, the removal of data blocks (based on gene-boundaries or on protein domains) with an IC = 0 and only keeping partitions having contributing sequences from all nine species, supermatrix A (based on protein domain data blocks) consisted of 370,877 amino acid sites and 1049 data blocks (328 Pfam-A domains, 161 clans, 560 voids) and supermatrix B (based on gene data blocks) comprised 323,917 amino acid sites with 894 data blocks (Additional file 8). PartitionFinder merged input data blocks into 338 meta-partitions for supermatrix A (protein domain-based) and 343 meta-partitions for supermatrix B (gene-based). The best fitting substitution models assigned to the meta-partitions were mostly LG4X and LG + G + F (Additional file 9).

**Fig. 7:**
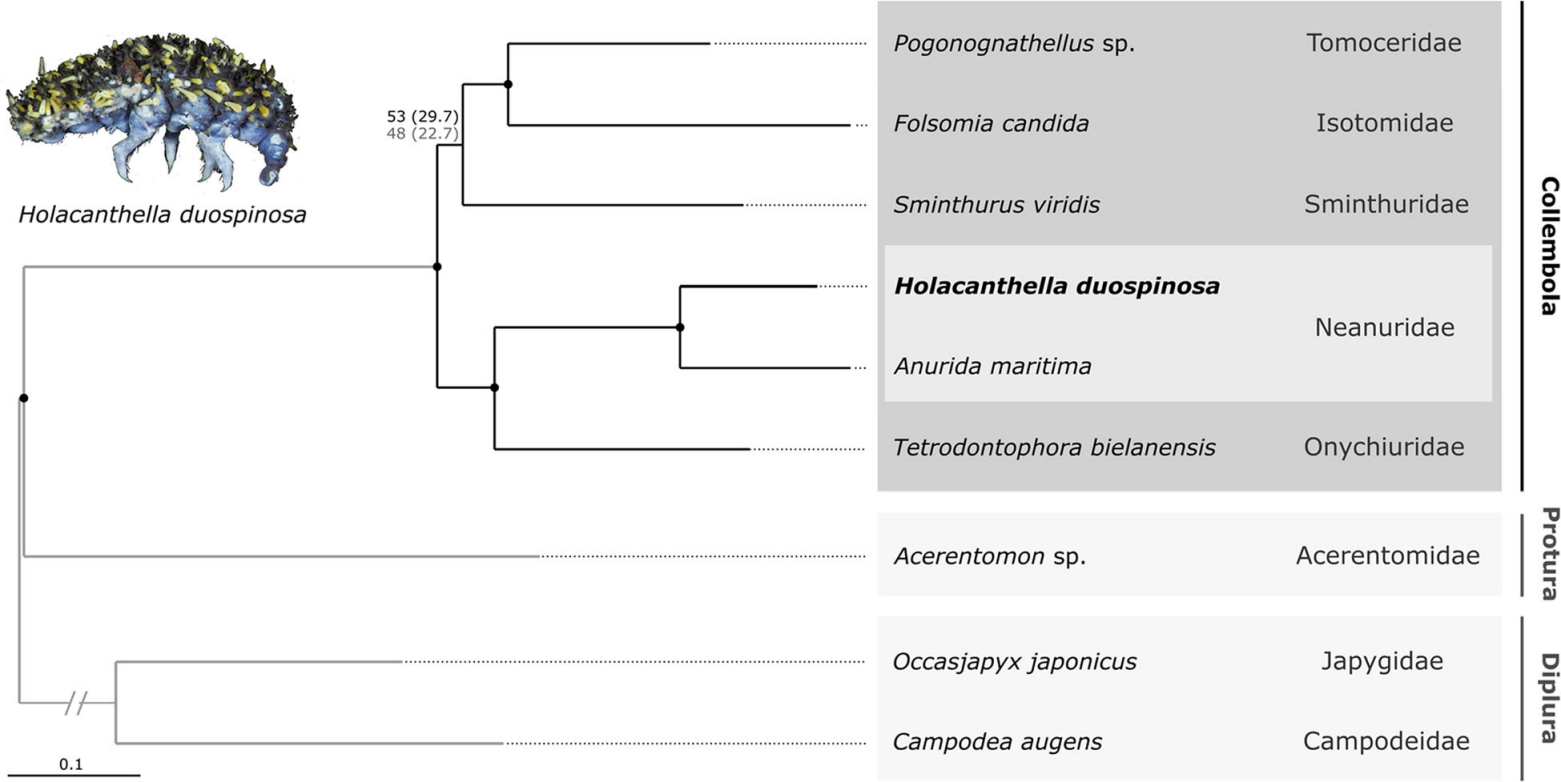
Best phylogenetic tree inferred with a Maximum Likelihood approach by IQtree (see Methods). Non-parametric bootstrap support was derived from 300 bootstrap replicates. The tree was rooted with Diplura. For both datasets, all inferred relationships revealed maximal support except for the placement of *Sminthurus viridis*: in black: bootstrap support for the dataset with domain-based meta-partitions (in parentheses support from the SH-LRT test), in grey: bootstrap support for the dataset with gene-based meta-partitions (in parentheses support from the aLRT test). Nodal black dots indicate maximal bootstrap and single branch test support. Photograph: Mark I. Stevens

Both datasets yielded a similar optimal tree with Collembola being monophyletic and *H. duospinosa* consistently placed as closest relative to *Anurida maritima* (Neanuridae). All clades show maximal support, except for the placement of *Sminthurus viridis* (Symphypleona, Sminthuridae) (Fig. 7). For supermatrix A (domain-based meta-partitions), we found one unique topology as displayed in Fig. 7. For supermatrix B (gene-based meta-partitions) we found two tree topologies whereas the trees with the better LogLH were similar to the unique topology of supermatrix A (40 out of 50 trees). The alternative topology from supermatrix B (10 out of 50 trees) placed *Sminthurus* as sister to all other springtails which contributes to the very low bootstrap support for the clade *Sminthurus* + (*Pogonognathellus* + *Folsomia*). Thus, the placement of *Sminthurus* as representative of Symphypleona remains ambiguous as found previously [2]. This issue will be addressed in future phylogenetic studies from the 1KITE consortium. The phylogenetic analysis of both data sets support the expected sister group relationship between *H. duospinosa* and *Anurida*.

## Conclusions

Our assembly of the giant Collembola, *Holacanthella duospinosa*, genome provides a new resource for understanding critical events in the evolutionary history of the arthropods and in particular Hexapoda. Previous phylogenomic studies have indicated that the Collembola likely diverged from Protura (cone-heads) in the Ordovician to Devonian [2]. Our phylogenetic reconstruction using more than 370,000 amino acids, supports a monophyletic Collembola with Protura as their sister group [2, 9]. Relationships among collembolan clades were not fully resolved with the ambiguous placement of Sminthuridae in the phylogeny.

Our data complement those of the *Orchesella cincta* genome, from Entomobryidae [10] and *Folsomia candida*, from Isotomidae [16]. This new collembolan genome helps fill a gap in the growing suite of arthropod genomes, especially those outside the hyperdiverse Insecta [18, 20]. Moreover, our assembly is high quality relative to many published arthropod genomes, as shown by the assembly quality statistics and the number of conserved BUSCO genes that were detected.

We focussed on several aspects of genome biology that underpin the evolutionary success of Hexapoda, including the diversity of chemosensory receptors, environmentally responsive DNA methylation, sex determination and the genomic structure of suites of key developmental genes. The substantial variation in the morphology and ecology of the Collembola provides a rich resource for exploring how the genome has evolved within this group. For *Holacanthella* in particular, their giant size relative to other collembolan species coupled with a number of unusual morphological features such as brightly coloured digitations and cuticular colouration [15] presents opportunities to investigate the origins of these traits.

## Methods

### DNA and RNA extraction and sequencing

Several individuals of *Holacanthella duospinosa* were collected from under rotting logs on the slopes of Hauturu-O-Toi (Little Barrier Island, 36.19 °S, 175.11 °E), an island in the Hauraki Gulf, near Auckland, New Zealand. We estimated the size of the genome to be 320 Mbp with flow cytometry using methods described previously [81]. Total genomic DNA was extracted from a single individual with the DNeasy kit (Qiagen) using the animal tissue protocol, and the addition of a 3-min incubation with 0.02 mg of RNase A after the digestion step and then centrifugation for 3 min at 12,100 g to remove any remaining material. We sequenced the genome of *H. duospinosa* using the Illumina HiSeq 2000 sequencing platform. The sequencing libraries consisted of three paired end (PE) libraries with insert sizes of 188 bp, 200 bp and 470 bp and two mate paired (MP) libraries with 3 kbp and 5 kbp insert sizes. The paired end libraries were prepared using the Illumina TruSeq RNA kit and the mate pair libraries using the Illumina TruSeq DNA kit. These libraries were run on two lanes of an Illumina HiSeq2000 at New Zealand Genomics Ltd., Dunedin. Total RNA was extracted from a separate individual using Trizol (Invitrogen) following manufacturer’s instructions for the TRIzol Plus RNA Purification Kit. Four RNA extractions were made from antennae, head, thorax and abdomen. These RNA extractions were used to prepare four mRNA libraries using the Illumina TruSeq RNA kit and run on a single lane of an Illumina HiSeq2000 at New Zealand Genomics Ltd., Dunedin, New Zealand.

### Genome and transcriptome assembly

The paired end (PE) reads were filtered for duplicate pairs, reads containing ambiguities (Ns), and then trimmed of adapter sequences and low quality ends using FastUniq (v1.1), PrinSeq (v0.20.3) and Cutadapt (v1.3), respectively [82–84]. Read pairs with at least one read less than 50 bp and unpaired reads (singletons) were discarded. Reads from the short-insert PE libraries (188, 200 and 470 bp) were decomposed into short sequences of length k (k-mer, with k = 17) using SOAPec (v2.03). These reads were then error corrected using ErrorCorrectReads.pl script from the ALLPATHS-LG (v46436) package [85] and the 188 bp and 200 bp PE libraries were merged into long single reads if a pair was detected with an overlap longer than 11 bp. A similar cleaning procedure was also applied to the two mate pair libraries. However, instead of error correcting, we retained 36 bases from the 5′ end of all cleaned reads in order to avoid disruption from the internal adapter sequences.

We used SOAPdenovo2 (vR223) [86] with a k-mer of 73 to perform the initial de novo assembly on reads from PE libraries. We then filled the gaps of the scaffolds using GapCloser (v1.12-r6) [86] and joined the resulting sequences with a standalone scaffolding program called SSPACE (v2.0) [87] using the same paired information. We then used SSPACE again to join the improved scaffolds into longer sequences with the two MP libraries and finally filled the gaps again with PE data.

We assessed the completeness of the assembled genome through three steps. First, to evaluate if the assembly has covered most of the sequencing reads, we mapped all PE reads back to the assembly using Bowtie2 (v2.2.0) [88]. Second, we mapped transcriptome RNA-Seq read pairs to the genome assembly in order to estimate how well the gene coding regions were assembled. Third, we estimated the completeness of the 1066 highly conserved Arthropoda genes (database: arthropoda_odb9) in the genome assembly using BUSCO (v2.0.1) [28]. Furthermore, the scaffolds were searched for homologues from GenBank nucleotide database (*nt*) using BLASTN (v2.2.28) [89] to determine whether contaminated sequences derived from microbes were present. The scripts from Assemblage (https://github.com/sujaikumar/assemblage) were used to assign the BLAST matches to different taxonomic categories.

We sequenced the *H. duospinosa* transcriptome to inform predicted gene models. The collembolan transcriptome included RNAseq libraries from the antennae, head, thorax and abdomen, sequenced together across two lanes of HiSeq 2000. The reads were pooled together for de novo assembly. Before reads were assembled, they were filtered and trimmed using a similar cleaning strategy to that applied to the genomic data, except the RNA-Seq reads were trimmed of 8 bases at the 5′ end before the rest of cleaning steps. The remaining high quality reads were then error corrected, before assembly using Trinity (r20140413p1) [90] with default options. The final transcriptome assembly was achieved after sequence redundancy was removed using CD-HIT (v3.1.1) [91] with a 95% identity threshold.

### Genome and transcriptome annotation and comparative analysis

We searched for and classified repeats using RepeatModeler (v1.0.8) [92] and PASTEClassifier (v1.0) [93]. The program RepeatMasker (v4.0.5) [94] was used to mask the genome assembly prior to annotation for protein coding genes. We performed structural gene annotation with MAKER2 (v2.31.3) [95] on the repeat-masked genome assembly, integrating transcripts from the transcriptome assembly and conserved Arthropoda protein sequences to correct the predicted gene models. The whole pipeline was divided into several steps. First, the program Augustus [96] was trained using 248 predicted protein models together with 150 complete protein-coding transcripts determined by TransDecoder from the Trinity transcriptome assembly [90]. The trained gene structure parameters were then used by MAKER2 to predict gene structures. Second, the homology evidence provided to MAKER2 included the assembled transcriptome set, 3028 conserved arthropod protein models, which we downloaded from OrthoDB (v7). For the annotation of specific genes, sequences were identified by BLAST searches on assembled transcriptomes and the genome assembly. Where similar transcripts could not be identified, gene models generated by FGENESH6 [97] were used to identify partial regions of coding sequence. We searched for candidate horizontal transfer events as genes identified from the scaffolds that also contain host (insect) genes. We assigned taxonomic identity to each gene model from the ‘blast_taxonomy_report.sl’ using ASSEMBLAGE (https://github.com/sujaikumar/assemblage).

For annotation of RNA coding genes we used the cmsearch program from INFERNAL (v1.1.1) and corresponding covariance models (CMs) from the Rfam database (v12.0) [98, 99]. All matches above the curated GA threshold were included. INFERNAL was selected as the predictions it makes are the most accurate for ncRNAs that have been identified to date [100]. In order to refine the annotation of tRNA genes we ran tRNA-scan (v1.3.1) [101]. This method also uses CMs to identify tRNAs. However, it also uses some heuristics to increase the search-speed and annotates the isoacceptor type of each prediction. It also has a method to infer whether predictions are likely to be functional or tRNA-derived pseudogenes. Rfam matches and the tRNA-scan results for families belonging to the same clan were then “competed” so that only the best match was retained for any genomic region [102].

Protein sequences of genes known to be associated with sex-determination, particularly in insects and *Daphnia* (Additional file 6)*,* were collected from UniProtKB and used as queries for TBLASTN searches against the *H. duospinosa* transcriptome and genome assemblies. The top BLAST hits with an E-value threshold of 1e^−05^ were retrieved and used as queries for reciprocal BLAST searches against the NCBI non-redundant protein database to confirm putative orthology. The relationships of the doublesex (*dsx*) and sex-lethal (*sxl*) sequences to other known orthologues were tested using phylogenetic approaches. Briefly, the protein sequences (putative ORF identified from the transcript) of *dsx* and *Sxl* were aligned against known orthologues of various insects, as well as some Crustacea and Chelicerata (Additional files 4 and 5) using the online version of Mafft (v7) [103] with scoring matrix BLOSUM45 and default settings. The phylogenies of *dsx* and *Sxl* were rooted at the Ixodida and Branchiopoda, respectively. Sequence alignments of chitinase homologues were created using the Mafft plugin within Geneious v10.0.3 [104]. Phylogenies were reconstructed from alignments of the sex determination and chitinase proteins with the PhyML plugin [105] within Geneious using the JTT + Γ substitution model and 1000 bootstrap replicates.

Genome-wide heterozygous sites were determined by calling variants between the two haploid components in the diploid genome. Reads used for genome assembly construction were mapped to the *H. duospinosa* genome assembly using Bowtie2 (v2.2.0) [88]. Paired end reads were maintained if both pairs are concordantly mapped on one scaffold (> 97%). Variant-calling was performed with SAMtools (v1.2) and BCFtools (v1.2) [106, 107]. We filtered variants with low quality (QUAL < 30) and/or with excessive mapping depth (DP > 250). The remaining variants were then assigned to gene coding regions using custom python scripts. Exons of length shorter than 30 bp were filtered due to high occurrence of spurious annotation. All the heterozygous genes were then assigned to Gene Ontology (GO) terms using Blast2GO (v2.8) [108]. Enriched GO terms were calculated with the same program using Fisher’s Exact Test with multiple testing correction of false discovery rate [109] less than 0.05.

Nucleotide and dinucleotide content of gene body sequences (both full-length and uniformly truncated to 1 kb) and 1 kb fragments of the whole genome sequence were calculated using a custom Perl script. CpG[o/e] was calculated using the formula CpG[o/e] = (N*CpG)/(C*G), where N is the length of the genomic region, CpG is the number of CpG sites in the regions, and C and G are the numbers of cytosines and guanines in the region [110]. Calculated CpG[o/e] values were plotted as frequency distributions in R (www.r-project.org). The number of components underlying these distributions was estimated in R using mclust [111] model-based clustering. The best fitting model was identified among several non-nested models using Bayesian information criteria (BIC).

### Phylogenetic analyses of whole transcriptomes

We inferred phylogenetic relationships from nine species (one proturan, two diplurans and six collembolans) including transcriptome data of eight species published previously [2] (current assembly version, see Additional file 7 and NCBI, 1KITE-Umbrella Bioproject ID 183205) and the transcriptome of *H. duospinosa* (Additional file 7). We first assigned assembled transcripts of each species to orthologous single copy genes (OGs) published by [2] using the orthology predicting programme Orthograph (v0.5.11) [112]. The published ortholog set comprises 1478 OGs and is based on 12 arthropod reference species (see [2], Table S3). Briefly, Orthograph generates profile hidden Markov models (pHMMs) from alignments of orthologous genes with a set of reference species. The pHMMs are then used to search transcript assemblies of query species for putative candidate orthologous sequences. Candidate sequences are then validated by a reciprocal BLAST using the official gene sets of reference species included in the ortholog set. Surviving hits are considered as ortholog transcript sequences. We chose a relaxed reciprocal BLAST search to any of the reference species. Other settings than default were “max-blast-searches” and “max-blast-hits” = 50; “extend-orf = 1”, and “substitute-u-with = X”. The latter avoids potential problems in downstream analyses because most programmes cannot handle selenocysteine (U). After summarising the results at the amino acid level, we masked all stop codons with “X”. Since we received no hit for two OGs from any of the query species and for another six OGs only hits for one species, we excluded these genes from further analyses. We then generated amino acid multiple sequence alignments (MSAs) for each OG (1470 OGs) using MAFFT L-INS-i (v7.123b) [103]. As described in [2], we checked each MSA for ambiguously aligned sequences (outliers) with the result of none being identified (see Result). For further downstream analyses we subsequently removed all sequences of the reference species from the alignments, leaving only sequences of the nine query taxa and deleted all columns containing only ‘X’ and/or ‘-’ (gaps).

We proceeded to design two datasets, one with partitions based on gene-boundaries and a second one with partitions based on protein domain-boundaries. For both datasets, we identified randomly similar aligned sites within each MSAs of each orthologous gene using a modified version of Aliscore (v1.2) [2, 9, 113], with the same settings described previously [2]. For the dataset based on gene-boundaries, we removed ambiguously aligned sections with the aid of Alicut (v2.3) [114], replaced terminal gaps by ‘X’, and concatenated masked MSAs into a supermatrix using FasConCat (v1.0) [115]. For the dataset based on protein domain-boundaries, we identified protein domains with the protein family database Pfam (v28, released 5 June 2015) [116], more specifically the Pfam-A pHMM library, following a procedure published previously [2] using the PfamScan software (v1.5, released 26 June 2015) [116] and HMMER (v3.1b1) (http://hmmer.org/). We then deleted ambiguously aligned sections from domain-based data blocks using the results of Aliscore and subsequently concatenated data blocks based on domain-boundaries into a supermatrix using custom Perl scripts. For each supermatrix, one with data blocks based on protein-domains (supermatrix A), the other with data blocks based on gene-boundaries (supermatrix B), we evaluated the information content (IC) of each data block with the software MARE (v0.1.2-rc) [117]. From both supermatrices, we removed data blocks with an IC = 0 and only kept data blocks for which all nine species were present.

For the selection of optimal meta-partitions and the best-fitting amino acid substitution models (see [2], Material and Methods, Section 3.6), we applied PartitionFinder (v2.0.0, prerelease 13) [118, 119] on both supermatrices in combination with RAxML (v8.2.3) [120]. We restricted the estimation of the best-fitting amino acid substitution model to LG [121], WAG [122], DMCUT [123], JTT [124], BLOSUM62 [125], each plus the alpha-shape parameter (+GAMMA) to account for among-site rate variation [126] and, in addition, listed models +Γ and using empirical base frequencies (+F). Moreover, we included the recently published free rate model LG4X [127] resulting in altogether 11 models. For the analyses we chose linked branch lengths and used the corrected Akaike information criterion (AICc, [128]) for final model selection. We applied the rcluster algorithm with the following settings: rcluster-max 10,000, rcluster-percent 100, all-states, min-subset-size 100, weights 1,1,0,1.

For maximum likelihood phylogenetic tree inference from both supermatrices, we applied IQTREE (v1.4.2) [129]. The search settings included 50 tree searches with the best meta-partition scheme and best-fitting model per meta-partition (option -spp), and using random starting trees for tree searches. For statistical support, we applied non-parametric bootstrap analyses (300 bootstrap replicates, partitioned bootstrapping). Finally, we plotted all bootstrap replicates on the ML tree with the best log LH value. We performed a SH-like approximate likelihood ratio test (see [105]) with 10,000 replicates on both data sets. We further checked how many unique topologies were present within the 50 inferred trees using the software Unique Tree (v1.9) (T. Wong, L. Jermiin, available upon request). For visualising and rooting the final tree with Diplura, we used Seaview (v4.2) [130]. We edited the tree graphically using Inkscape (v0.91) (https://inkscape.org).

## Additional files

Additional file 1: BLAST results from gene models. (XLSX 1068 kb)

Additional file 2: Single nucleotide variants. (XLSX 775 kb)

Additional file 3: Candidates for horizontal gene transfer from bacteria and fungi into *Holacanthella duospinosa*. (XLSX 24 kb)

Additional file 4: Nexus alignment of *dsx* transcripts from across arthropods. (TXT 12 kb)

Additional file 5: Nexus alignment of *sxl* transcripts from across arthropods. (TXT 4 kb)

Additional file 6: Table of putative sex determination genes. (DOCX 19 kb)

Additional file 7: Bioproject IDs, TSA accession numbers and number of orthologous hits of the nine specimens used in the phylogenetic analysis. (XLSX 12 kb)

Additional file 8: Number of amino acid sites and data blocks of supermatrices A and B at different steps in the analysis. For the domain-based supermatrix A, data blocks are composed of Pfam-A domains, clans and voids. (XLSX 5 kb)

Additional file 9: The best fitting substitution models and number of meta-partitions for supermatrix A (domain-based) and supermatrix B (gene-based) according to PartitionFinder. (XLSX 5 kb)

## Abbreviations

Bp: Base pair
GO: Gene ontology
GR: Gustatory receptor
HGT: Horizontal gene transfer
IR: Ionotropic glutamate receptor
Kbp: Kilo base pair
lncRNA: Long non-coding RNA
LTR: Long transcribed repeat
Mbp: Mega base pair
miRNA: Micro RNA
MSA: Multiple sequence alignment
ncRNA: Non-coding RNA
OR: Odorant receptor
SINE: Short interspersed element
snoRNA: Small nucleolar RNA
snRNA: Small nuclear RNA
SSU rRNA: Small subunit RNA
