# Supplementary material for: Analysis of the genome of the New Zealand giant collembolan (Holacanthella duospinosa) sheds light on hexapod evolution
Source: BMC Genomics. 2017 Oct 17;18:795. doi: 10.1186/s12864-017-4197-1 (PMC5644144; doi:10.1186/s12864-017-4197-1)
Supplement: Supplementary file 6 — Table of putative sex determination genes. (DOCX 19 kb) [file 12864_2017_4197_MOESM6_ESM.docx]

Table SX. Putative *Holacanthella* *duospinosa* sex determining genes. Transcript ID refers to transcripts in the *Holacanthella* NCBI accession GFPE00000000. Protein domains were detected with NCBI Conserved Domains. Genes that matched a transcript in the initial Blast search but were not confirmed by reciprocal Blast are shaded in grey.

| **Gene name** | **Function** | **Transcript ID** | **Reciprocal BLAST** | **Domains present** |
| --- | --- | --- | --- | --- |
| doublesex (dsx) | Controls somatic sexual differentiation | c36538_g1_i2 | doublesex | DM and DSX_dimer |
| fruitless (fru) | Role in male courtship behaviour and sexual orientation | c21432_g1_i2 | fruitless | BTB domain |
| daughterless (da) | Maturation of follicle cells during egg chamber morphogenesis | c36082_g1_i1 | WD-repeat containing protein 37 | WD40 domain |
| doublesex-mab related 99B (dmrt99B) | Transcription factor activity | c4199_g1_i1 | DMTRA2 (=dmrt99B) | DM |
| sexlethal (sxl) | Involved in splicing transformer | c26448_g1_i2 | sex-lethal | RRM2_SXL, RRM1_HuC |
| transformer-2 (tra2) | Female-specific splicing and/or polyadenylation of doublesex pre-mRNA (and fruitless) | c17528_g1_i1 | transformer-2 | RRM_TRA2B |
| transformer (tra) | Female-specific splicing and/or polyadenylation of doublesex pre-mRNA (and fruitless) | no hits |  |  |
| feminizer (fem) | Homolog of tra (in *Apis*) | no hits |  |  |
| Complementary sex determiner (cds) | Determines processing of fem | no hits |  |  |
| Sans fille (snf) | Involved in the female-specific splicing of the late sex-lethal pre-mRNA | c5489_g1_i1 | U1 small nuclear ribonucleoprotein A (=snf) | RRM1_U2B, RRM2_SNF |
| virilizer (vir) | Involved in the female-specific splicing of the late sex-lethal and tra pre-mRNA | c37529_g1_i4 | virilizer |  |
| intersex (ix) | Interacts with the female-Dsx variant: controls female terminal differentiation | c32122_g1_i2 | mediator of RNA polymerase II transcription subunit 29 (= ix) | Med29 |
| hermaphrodite (her) | Early sxl activation and involved in female and male terminal differentiation | c39478_g1_i3 | zinc finger protein 135-like | zf-H2C2_2 |
| female-lethal- 2-d (fl(2)d) | Female-specific splicing of the late sxl and tra pre-mRNA | c30038_g1_i2 | female-lethal- 2-d | Mplasa_alph_rch |
| sisterless A (sisA) | Forms X/A signal in two drosophilids | no hits |  |  |
| achaete-scute complex protein T4 (sc) | Forms X/A signal in one drosophila species | c28000_g1_i1 | achaete-scute complex protein T4 | HLH |
| deadpan (dpn) | Represses sxl transcription | c21442_g1_i1 | deadpan | ORANGE, HLH |
| extra-macrochaetae (emc) | Primary response to X:A ratio | c27046_g2_i2 | extra-macrochaetae | HLH |
| Runt (run) | Involved in activation of sxl | c98276_g1_i1, | runt-related transcription factor 1-like | Runt |
| maleless (mle) | Involved in dosage compensation | c39299_g5_i2 | dosage compensation regulator | 2x DSRM, 2x HrpA |
| males-absent on the first protein (mof) | Involved in dosage compensation | c35798_g1_i1 | histone acetyltransferase KAT8 (=mof) | MOZ/SAS, Tudor-k |
| male-specific lethal-3 (msl-3) | Involved in dosage compensation | c34608_g1_i1 | male-specific lethal-3 | 2x MRG |
| male-specific lethal-1 (msl-1) | Involved in dosage compensation | c35786_g1_i1 | hybrid signal transduction protein dokA-like, serine/threonine-protein kinase pakD-like, Male-specific lethal 1 like protein | PEHE |
| E3 ubiquitin-protein ligase (msl-2) | Involved in dosage compensation | No hits |  |  |
| boule (bol) | Plays central role in spermatogenesis | c5367_g1_i1 | trithorax group protein osa-like isoform X2 | RRM_SF |
| testis-specific zinc finger protein (topi) | Required for male meiotic division and spermatid differentiation | c32771_g1_i1 | zinc finger protein 275-like | Tryp_SPc, Zn-finger |
| outstretched (os) | Required for sxl expression | No hits |  |  |
| chromosomal serine/threonine-protein kinase  (JIL-1) | Involved in dosage compensation | c21448_g1_i1 | ribosomal protein S6 kinase alpha-5-like isoform X2 | 2x S_TKc |
